# Persistence of passive immunity in calves receiving colostrum from cows vaccinated with a live attenuated lumpy skin disease vaccine and the performance of serological tests

**DOI:** 10.3389/fvets.2024.1303424

**Published:** 2024-05-21

**Authors:** Amarin Rittipornlertrak, Wittawat Modethed, Kanokwan Sangkakam, Anucha Muenthaisong, Paramintra Vinitchaikul, Kittikorn Boonsri, Kidsadagon Pringproa, Veerasak Punyapornwithaya, Khwanchai Kreausukon, Nattawooti Sthitmatee, Tawatchai Singhla

**Affiliations:** ^1^Faculty of Veterinary Medicine, Chiang Mai University, Chiang Mai, Thailand; ^2^Laboratory of Veterinary Vaccine and Biological Products, Faculty of Veterinary Medicine, Chiang Mai University, Chiang Mai, Thailand; ^3^Research Center for Veterinary Biosciences and Veterinary Public Health, Faculty of Veterinary Medicine, Chiang Mai University, Chiang Mai, Thailand; ^4^Chiang Mai Livestock Office, Department of Livestock Development, Ministry of Agriculture and Cooperative, Chiang Mai, Thailand; ^5^Office of Research Administration, Chiang Mai University, Chiang Mai, Thailand

**Keywords:** lumpy skin disease, newborn calves, passive immunity, virus neutralization test, enzyme-linked immunosorbent assay

## Abstract

This study aimed to determine the persistent duration of maternal immunity against lumpy skin disease virus (LSDV) in dairy calves born from vaccinated cows using a virus neutralization test (VNT). The performance of the VNT and an in-house-ELISA test was also determined. Thirty-seven pregnant cows from 12 LSD-free dairy farms in Lamphun province, Thailand were immunized with a homologous Neethling strain-based attenuated vaccine and calved from December 2021 to April 2022. Blood samples from dam-calve pairs were collected within the first week after calving. Subsequently, blood samples were taken from the calves at monthly intervals over a period of 4 months and tested for the humoral immune response using a VNT. The calf sera were also tested with an in-house ELISA test to estimate the accuracy of both tests using a Bayesian approach. For the results, antibodies against LSDV can persist in cows for 4–9 months post-vaccination. Moreover, neutralizing antibodies and LSDV-specific antibodies against LSDV were detected in the majority of calves (75.68%) during the first week after colostrum intake. However, the percentage of seropositive calves declined to zero by day 120, with seropositivity dropping below 50% after day 60. Only a small number of seropositive calves (approximately 13.51%) were observed on day 90. These findings indicated that passive immunity against LSDV can last up to 3 months. The median of posterior estimates for sensitivity (Se) and specificity (Sp) of the VNT were 87.3% [95% posterior probability interval (PPI) = 81.1–92.2%] and 94.5% (95% PPI = 87.7–98.3%), respectively. The estimated Se and Sp for the ELISA test were 83.1% (95% PPI = 73.6–92.6%) and 94.7% (95% PPI = 88.4–98.5%), respectively. In conclusion, this study illustrates the transfer and persistence of maternal passive immunity against LSDV to calves under field conditions. This highlights a potential three-month vaccination gap in calves born from vaccinated cows, while an in-house ELISA test can be used as an ancillary test for LSDV immune response detection. However, further research is required to assess the vaccination protocols for calves as young as 2 months old to precisely determine the duration of maternal immunity.

## Introduction

Lumpy skin disease (LSD) is an important transboundary viral disease in cattle and buffalo caused by the lumpy skin disease virus (LSDV) belonging to the genus *Capripoxvirus*, family *Poxviridae*. Blood-sucking insects, such as stable flies, mosquitoes, and ticks, are thought to play a role in disease transmission as mechanical vectors (1–4). The mobility rate of LSDV infection varies from 5 to 45%, but in some cases, it can reach up to 100%. However, the mortality rate is relatively low, at around 10% (and occasionally up to 40%) (5). Given its rapid spread and significant economic impact, LSD is classified as a notifiable disease by the World Organization for Animal Health (WOAH) (6). It is an endemic disease prevalent across Africa and the Middle East (3). Since 2015, LSD has been spreading to Europe (7). In 2019, LSDV was confirmed in East Asia and South Asia, including China (8), Bangladesh (9), and India (10). LSDV was subsequently detected in Southeast Asia in 2020, including Vietnam (11) and Myanmar (12). In 2021, LSD was eventually established in Thailand (13), leading to a nationwide outbreak with morbidity and mortality rates of 40.5 and 1.2%, respectively (14).

In response to the first LSD outbreak in Thailand, the Department of Livestock Development (DLD) implemented considerable control measures, including strict quarantine and movement control, zoning, surveillance outside/within the protection zone, vector control, and disinfection in the outbreak area (15). A million doses of live attenuated vaccines [Lumpyvax™ (MSD, Pretoria, South Africa)] and MEVAC™ (Kemin, Cairo, Egypt) have been imported, and extensive vaccination campaigns have been implemented on the cattle population in the country to control disease distribution (15, 16). According to the manufacturers, calves born to vaccinated cows should be immunized at the age of 6 months, followed by annual boosters thereafter. However, based on field observation, it is likely that calves aged less than 3 months can be clinically infected with LSDV before receiving the vaccine. This indicates that the timing of the vaccination should be revised. Therefore, it is essential to measure the persistence of maternal passive immunity against LSDV in calves in order to elucidate and determine the optimal vaccination program for calves in affected countries.

To detect the humoral immune response to LSDV in animals, a virus neutralization test (VNT) has been suggested by WOAH as the only validated serological test available. However, VNT is time-consuming and costly. Moreover, it has been reported that the sensitivity (Se) of this technique is low and the specificity (Sp) quite high, ranging from 74.0–83.0 and 95.0–99.0%, respectively (17–19). To enhance the detection performance of antibodies against LSDV, an ELISA test has been developed due to its advantages such as rapidity, high Se and Sp, and cost-effectiveness (20–22). Likewise, during LSD outbreaks in Thailand, an in-house ELISA test was developed to detect antibodies against LSDV in cattle, and only one report on the accuracy of the test has suggested that the Se and Sp of the ELISA test were 94.9% (86.7–99.7%) and 89.8% (75.9–99.3%), respectively (22). Therefore, information on the characteristics of both VNT and the in-house ELISA tests needs further estimation.

The aim of this study was to evaluate the duration of maternally derived neutralizing antibodies against LSDV in calves born from cows vaccinated with a homologous Neethling strain-based live attenuated vaccine in field conditions using a virus neutralization test to design a vaccination program for calves. Furthermore, the performance of serological tests including VNT and in-house ELISA test was also determined.

## Materials and methods

### Area of study

This study was conducted in 12 LSD-free dairy farms in Ban Thi District, Lamphun province, Thailand. All dairy cattle herds received subcutaneous vaccinations with live attenuated vaccine (LUMPYVAC®; Vetal Animal Health Product SA; Adiyaman, Turkey) approximately 4–9 months before the start of the study.

### Animals and sampling

Thirty-seven clinically healthy dams and their calves were included in the study. Cows calved from December 2021 to April 2022, and the calves were separated from their dams immediately after birth. All newborn calves were fed with 2–3 L of fresh colostrum from their dams within 6–12 h after birth, following the farm’s protocol without intervention. Blood samples were collected from dams via coccygeal venipuncture and their calves via jugular venipuncture within the first week after calving, following the initial colostrum intake. Subsequently, blood samples were collected at monthly intervals for a total of 4 months (30, 60, 90, and 120 days post-vaccination). The serum samples were allowed to clot at room temperature and then centrifuged at 1,000 × *g* for 10 min. These sera were stored at −80°C until further examination.

### Virus isolation and cultivation

The local LSDV field strain was obtained from the skin nodules of infected cattle in Lampang Province. A 10% skin tissue suspension was filtered through a 0.45 μm filter. The filtered sample was then inoculated onto the Madin–Darby bovine kidney (MDBK) cell line (CCL-22™ ATCC®, Manassas, VA, United States) in Dulbecco’s Modified Eagle’s Medium (DMEM; Wisent Bio Products St-Bruno, QC, Canada) supplemented with 1X antibiotic-antimycotic (100 units/mL of penicillin, 100 μg/mL of streptomycin, and 0.25 μg/mL of amphotericin B; GibcoTM, Life Technologies Waltham, MA, United States) and 10% fetal calf serum (FCS, GibcoTM) in a six-well plate. The cells were then incubated at 37°C in a 5% CO_2_ incubator for 7 days. Once the cytopathogenic effect (CPE) including cell membrane retraction, cell rounding, and margination of the nuclear chromatin was observed, the cell pellets were harvested and confirmed by PCR (23). Titration of virus stocks was performed using the Spearman-Kärber method (24).

### Virus neutralization test

The VNT was performed as previously described by Samojlović et al. (25). Briefly, VNT was performed in commercial flat-bottomed 96-well microtiter plates. Bovine sera, including control sera, were initially inactivated at 56°C for 30 min. A two-fold serial dilution of sera from 1:2 to 1:256 was then prepared in DMEM. Subsequently, 50 μL of the diluted serum was mixed with 50 μL of the local LSDV strain (100 TCID50, in DMEM). The plates were incubated at 37°C for 1 h. Then, 100 μL of MDBK cell suspension (with a concentration of 20,000 cells/mL) was added to each well containing the serum/virus mixture. The plates were then incubated in a 5% CO_2_ environment at 37°C for 3 days, with daily examination for the presence of cytopathic effects (CPE) using an inverted microscope. The infected bovine serum and colostrum-deprived neonatal calf serum were classified as positive and negative controls, respectively. Neutralizing antibody (NAb) titer at dilutions of ≥1:2 was considered positive. The positive and negative control sera used in this study were obtained from a previous study (22). The positive control serum originated from LSDV-infected cattle that was confirmed by PCR during the outbreak in Thailand in 2021. Furthermore, the serum was also tested seropositive with both a commercial ELISA kit (ID Screen® Capripox Double Antigen Multi-species by IDvet Innovative Diagnostics, France) (S/P ratio ≥ 30%) and an in-house ELISA (OD value ≥0.067). The negative control serum was colostrum-deprived neonatal calf serum which was also tested seronegative by both the commercial ELISA kit (S/P ratio < 30%) and the in-house ELISA (OD value <0.067). Additionally, the selected control sera must also meet specific VNT titer criteria ranging from 1:8 to 1:32 for positive control serum and less than 1:2 for negative control serum. Remarkably, the seropositivity and seronegativity of control sera were also confirmed by VNT. Furthermore, the back-titration of the 100TCID_50_ virus used in each test run was performed in four tenfold dilutions. The virus titer in back titration should be within 30 and 300 TCID_50_ according to the previous study (25).

### In-house enzyme-linked immunosorbent assay

The detection of LSDV-specific antibodies from investigated animals was performed using an in-house ELISA test as described by Sthitmatee et al. (22). Briefly, the LSDV coating antigen was prepared from the local LSDV strain. The virus particles were purified through 24–40% sucrose step gradients. After ultracentrifugation at 14,000 rpm for 40 min at 4°C, the purified viral band between the 32 and 36% sucrose layers was carefully pipetted and transferred to a new ultracentrifuge tube. The purified virus was pelleted and resuspended in 1 mL of cold 10 mM Tris–HCl, pH 8.0. The purity was checked by sodium dodecyl-sulfate polyacrylamide gel electrophoresis (SDS-PAGE). The purified viral particles were then inactivated with 0.3% formalin buffer to produce the LSDV coating antigen. The ELISA reaction was performed in a 96-well immunoplate (Nunc-Immuno™ MaxiSorp™; Sigma-Aldrich, St. Louis, MO, United States). The ELISA plates were coated with the LSDV antigen concentration of 1 TCID50/well in 0.05 M carbonate buffer (pH 9.6) and incubated at room temperature for 1 h. The plates were washed three times with washing buffer (PBST; 0.05% Tween20 in 0.01 M PBS, pH 7.2) and blocked with 100 μL per well of blocking buffer (1% bovine serum albumin in 0.01 M PBS, pH7.2) for 1 h at room temperature. After three washings with PBST, the sample sera (1:500) were diluted in blocking buffer and added to each well in duplication. The plates were incubated for 1 h at room temperature. After incubation, the plates were washed as previously described. The goat anti-bovine immunoglobulin G (IgG) labeled with horseradish peroxidase (HRP) (KPL, Gaithersburg, MD, United States) (1:10,000) diluted in blocking buffer was added and the plates were then incubated at room temperature for 1 h. After being washed three times with the washing buffer, the color reaction was developed using 3,3′,5,5′-tetramethylbenzidine (KPL). The plates were then incubated at room temperature in the dark for 15 min. The reaction was terminated by adding 50 μL of 3 M H_2_SO_4_. The optical density at a wavelength of 450 nm (OD450) was measured using an automatic ELISA plate reader (AccuReader; Metertech, Taipei, Taiwan). The calculated cut-off value was 0.067 and positive.

### Statistical analysis

Descriptive analysis was used to explain the persistence of passive immunity against LSDV in calves born from vaccinated dams. The agreement between VNT and in-house ELISA results was evaluated using Cohen’s kappa analysis. The results were interpreted based on kappa values (0–1), including slight (0–0.20), fair (0.21–0.40), moderate (0.41–0.60), substantial (0.61–0.80), and almost perfect (0.80–1.0) agreement (26). The characteristics of the VNT and in-house ELISA test were estimated using Bayesian latent class analysis due to the lack of a perfect reference test and unknown disease status as described elsewhere (27, 28). The Bayesian model of a conditionally dependent model was inferred in a single population because the principles of both tests being based on humoral immunity detection, and the animals were raised in the same region. The prior information on the Se and Sp of the two tests and the disease prevalence was derived from previous reports and modeled as beta distributions as shown in Table 1 (18, 21, 22, 25). Bayesian modeling was performed in JAGS 4.3.0 via the rjags and r2jags packages from R version 4.1.2 (29–31). The first 10,000 iterations were discarded as a burn-in phase, and posterior distributions were analyzed at 100,000 iterations of the model. A complete model and R codes are provided in the Supplementary material 1. The final model was tested for convergence using the Gelman-Rubin diagnostic plot visual inspection via three sample chains with different initial values as demonstrated in the Supplementary material 2 (32). To evaluate the influence of priors on posterior distributions, sensitivity analysis of the final model was performed by replacing each prior with a non-informative, uniform 0–1 distribution (28). More than 25% of changes in the model were considered as the appreciable effects of priors. The assumption of the conditionally dependent model was then tested by comparing the deviance information criteria (DICs) of the models with and without the covariance term. The final model was selected according to the lowest DIC (28, 32, 33).

**Table 1:** Prior estimates for mode and 95% credibility interval (CI) for sensitivity and specificity values of virus neutralization test and in-house ELISA test and prevalence of the disease (%).

Diagnostic tests	Parameters	Mode	95% CI^a^	Reference
VNT^b^	Sensitivity	83.0	>75.0	(18)
	Specificity	97.0	>95.0	(18)
ELISA^c^	Sensitivity	95.0	>87.0	(22)
	Specificity	90.0	>76.0	(22)
Disease prevalence		66.0	<83.0	(22)

^a^95% lower or upper credibility interval bound. ^b^Virus neutralization test. ^c^In-house enzyme-linked immunosorbent assay.

## Results

### Evaluation of humoral immune response in cows

Regarding the VNT, the range of the virus titer after back-titration of the diluted stock was approximately 80–200 TCID_50_. The antibody titers detected by VNT and ELISA varied in vaccinated cows, as shown in Table 2. Out of 37 cows, the neutralizing antibody titers and the ELISA titers were detected in 28 (75.7%) and 27 (73%) after 4–9 months of vaccination, respectively. Four cows were serologically positive by VNT but negative by ELISA. In contrast, three cow serum samples were serologically negative by VNT but positive by ELISA. Seropositivity and seronegativity were detected by both VNT and ELISA in 24 (64.9%) and 6 (16.22%) out of 37 cows, respectively (Table 3). It is possible that most of these 24 seropositive cows could transfer their maternal antibodies to their calves, except one (#9). Surprisingly, three of the six seronegative cows (#2, 3, 37) may have seropositive calves detected by either VNT or ELISA.

**Table 2:** Humoral immune response in dam-calf pairs determined by VNT and ELISA.

ID.	Cows		Calves									
	D7		D7		D30		D60		D90		D120	
	VNT	ELISA	VNT	ELISA	VNT	ELISA	VNT	ELISA	VNT	ELISA	VNT	ELISA
1	N	0.08	N	0.07	N	0.048	N	0.065	N	0.049	N	0.057
2	N	0.064	1:4	0.087	1:16	0.179	N	0.053	N	0.058	N	0.058
3	N	0.056	N	0.054	N	0.083	N	0.061	N	0.051	N	0.065
4	1:32	0.106	1:32	0.117	1:4	0.074	N	0.053	N	0.051	N	0.066
5	1:2	0.079	1:2	0.07	N	0.052	N	0.049	N	0.053	N	0.053
6	1:2	0.058	1:2	0.178	1:4	0.0895	1:4	0.053	N	0.053	N	0.054
7	1:8	0.123	1:8	0.076	1:8	0.1477	1:2	0.051	N	0.062	N	0.053
8	1:8	0.082	1:16	0.2465	1:8	0.132	1:8	0.113	1:4	0.0678	1:2	0.051
9	1:2	0.082	N	0.064	N	0.59	N	0.051	N	0.056	N	0.054
10	1:2	0.082	1:2	0.098	N	0.064	N	0.047	N	0.052	N	0.062
11	1:2	0.057	N	0.046	N	0.049	N	0.048	N	0.058	N	0.053
12	1:4	0.067	1:2	0.127	1:2	0.119	1:2	0.1	N	0.052	N	0.056
13	N	0.064	N	0.058	N	0.059	N	0.05	N	0.055	N	0.054
14	1:64	0.079	1:2	0.067	1:8	0.071	1:4	0.069	1:2	0.052	N	0.052
15	1:8	0.15	1:4	0.099	1:4	0.071	1:16	0.082	1:8	0.069	N	0.048
16	1:8	0.082	1:16	0.115	1:4	0.077	1:4	0.097	1:4	0.069	N	0.053
17	1:16	0.108	1:16	0.093	1:4	0.695	1:2	0.049	N	0.055	N	0.061
18	1:16	0.096	1:2	0.056	N	0.59	N	0.053	N	0.054	N	0.56
19	1:32	0.188	1:32	0.089	1:8	0.0725	1:4	0.048	1:2	0.051	N	0.066
20	1:64	0.265	1:8	0.1	1:2	0.63	N	0.065	N	0.05	N	0.058
21	1:2	0.069	1:2	0.083	N	0.07	N	0.63	N	0.055	N	0.048
22	N	0.057	N	0.59	N	0.051	N	0.061	N	0.048	N	0.065
23	1:128	0.391	1:64	0.373	1:16	0.175	1:4	0.075	1:2	0.054	N	0.064
24	1:16	0.085	1:16	0.094	1:8	0.086	1:4	0.675	1:4	0.065	N	0.063
25	1:16	0.074	1:2	0.075	1:16	0.15	1:2	0.066	N	0.054	N	0.055
26	N	0.65	N	0.06	N	0.064	N	0.065	N	0.058	N	0.045
27	N	0.074	1:4	0.07	1:2	0.046	N	0.048	N	0.49	N	0.05
28	1:2	0.086	1:4	0.052	1:8	0.101	1:2	0.067	N	0.63	N	0.057
29	1:4	0.072	1:8	0.089	1:4	0.08	N	0.49	N	0.057	N	0.048
30	1:2	0.078	1:4	0.069	1:2	0.05	N	0.06	N	0.054	N	0.046
31	1:8	0.069	1:16	0.083	1:4	0.067	1:2	0.056	N	0.052	N	0.051
32	1:4	0.066	1:2	0.073	1:4	0.069	1:8	0.089	1:4	0.069	1:4	0.049
33	1:8	0.069	1:4	0.08	1:4	0.074	1:8	0.086	1:4	0.069	N	0.05
34	N	0.075	1:4	0.071	1:4	0.048	N	0.05	N	0.066	N	0.048
35	1:2	0.065	1:4	0.072	1:2	0.063	N	0.066	N	0.059	N	0.055
36	1:2	0.122	1:4	0.153	1:8	0.117	N	0.063	N	0.061	N	0.054
37	N	0.064	1:2	0.17	N	0.5	N	0.066	N	0.053	N	0.044

ELISA ≥ 0.067 and VNT ≥ 1:2 are considered as seropositive. N: negative.

**Table 3:** Number of seropositive and seronegative animals determined by VNT and ELISA.

		Cows	Calves				
		D7	D7	D30	D60	D90	D120
VNT^a^	Positive	28	30	25	16	9	2
	Negative	9	7	12	21	28	35
ELISA^b^	Positive	27	29	21	10	5	0
	Negative	10	8	16	27	32	37
VNT/ELISA	Positive/Positive	24	28	19	10	5	0
	Negative/Negative	6	6	10	21	28	35
	Positive/Negative	4	2	5	5	4	2
	Negative/Positive	3	1	3	1	0	0

^a^Virus neutralization test. ^b^In-house enzyme-linked immunosorbent assay.

### Determination of persistence of maternally passive immunity in calves

**Figure 1:**
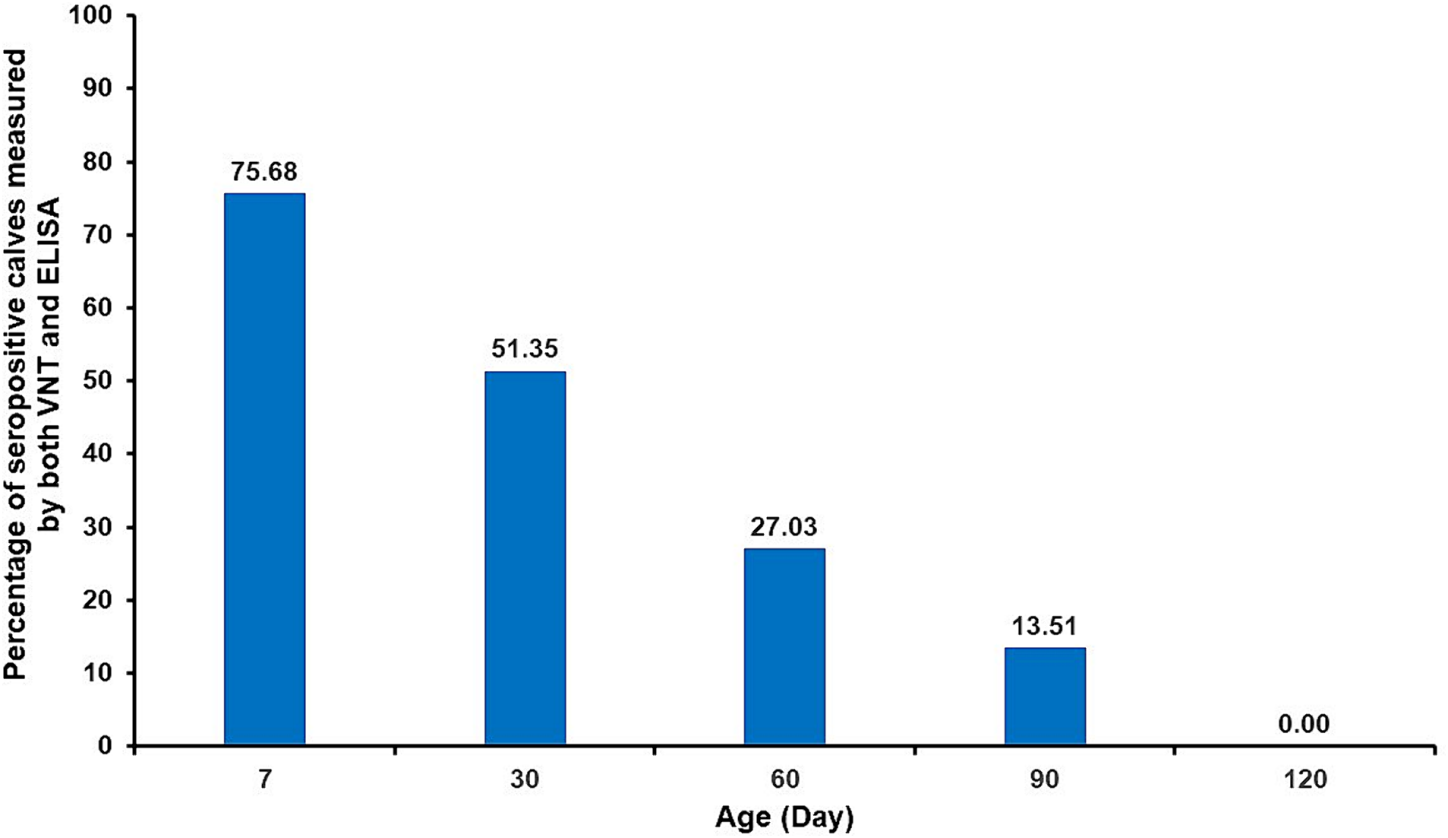
Percentage of seropositive calves against LSDV after colostrum intake until Day 120.

During the first week after their initial colostrum intake, 28 (75.7%) of the 37 calves were seropositive, whereas six (16.22%) calves were seronegative in both VNT and ELISA (Table 3). As shown in Figure 1, the percentage of seronegative calves gradually decreased from the first week after parturition to 13.51% on day 90 and to zero on day 120. From the VNT results obtained during the first week, it was observed that the majority of calves with detectable neutralizing antibodies (28 out of 30 seropositive calves) received colostrum from seropositive cows as determined by either VNT or ELISA. Nevertheless, it is worth noting that the other two calves (#2 and 37) tested positive for NAb, although their cows tested negative. Conversely, undetectable neutralizing antibodies were observed in seven out of 37 calves. Four of these seven calves were born to cows, confirmed as seronegative. However, the other three calves (#1, 9, and 11) were born to cows determined to be seropositive based on either ELISA or VNT. The results of the NAb titer on day 30 showed that the NAb titer of 1:2 did not provide detectable neutralizing antibodies until day 60, except for calf #12. The detailed results of passive transfer LSDV-specific antibodies are presented in Table 2.

### Performance of virus neutralization test and in-house ELISA test for the detection of antibodies against LSDV

Out of 222 serum samples, 108 (48.6%) and 93 (41.9%) samples were identified as positive by VNT and in-house ELISA tests, respectively. Twenty-three samples were positive by VNT and negative by ELISA, whereas eight samples were negative by VNT and positive by ELISA, as shown in Table 4. The VNT and ELISA results exhibited substantial agreement (*k* = 0.72).

**Table 4:** Cross-classified test outcomes for antibody against LSDV detection in 222 serum samples from virus neutralization test (VNT) and in-house ELISA test.

Diagnostic test results		ELISA^a^		Total
		Positive	Negative	
VNT^b^	Positive	85	23	108
	Negative	8	106	114
	Total	93	129	222

^a^In-house enzyme-linked immunosorbent assay. ^b^Virus neutralization test.

The posterior estimate for VNT-Se was higher than its priors, with a median of 87.3% [95% posterior probability interval (PPI) = 81.1–92.2%], and the posterior estimate for VNT-Sp lower than its priors, with a median of 94.5% (95% PPI = 87.7–98.3%). On the other hand, the estimated Se for the ELISA test was lower than its priors, with a median of 83.1% (95% PPI = 73.6–92.6%), whereas the estimated Sp for the ELISA was slightly lower than its priors with a median of 94.7% (95% PPI = 88.4–98.5%). The estimate for the disease prevalence was lower than the prior value, with a median of 51.2% (95% PPI = 42.2–60.2%). The posterior estimates of both serological tests and the true disease prevalence are shown in Table 5.

**Table 5:** Posterior estimates for characteristics of the virus neutralization test and in-house ELISA test and the true disease prevalence (%).

Diagnostic tests	Parameters	Median (%)	95% PPI^a^ (%)
VNT^b^	Sensitivity	87.3	81.1–92.2
	Specificity	94.5	87.7–98.3
ELISA^c^	Sensitivity	83.1	73.6–92.6
	Specificity	94.7	88.4–98.5
	Prevalence	51.2	42.2–60.2

^a^95% PPI = 95% posterior probability interval. ^b^Virus neutralization test. ^c^In-house enzyme-linked immunosorbent assay.

After checking for model convergence by visually inspecting the Gelman-Rubin diagnostic plots, the final model exhibited the proper convergence, and the autocorrelation was eliminated after the burn-in phase. Additionally, the result of the sensitivity analyses of the final model indicated robustness because there was no appreciable effect (a median value change >25%) in the posterior estimates of all parameters (the Se and Sp of both tests and the disease prevalence) after each prior being replaced by non-informative distributions.

For the assumption of the final model testing, the conditionally dependent model, with a covariance term between the VNT and ELISA test, showed a lower DIC value than the conditionally independent model (27.9 vs. 30.6, respectively). Thus, the conditionally dependent model was preferred as the final model.

## Discussion

This study evaluated the duration of passive transfer immunity against LSDV in calves after consuming colostrum derived from their dams that were vaccinated with a live attenuated vaccine in field conditions without the intervention of colostrum management. The defense mechanisms against disease in newborn calves are not fully developed. Hence, passive transfer immunity is crucial, offering initial protection through colostrum until the calf’s own immune system matures completely (34).

Regarding antibody titers, WOAH has documented that the humoral immune response against Capripoxvirus can remain detectable for approximately 7 months (35). In the present study, some cows seroconverted to a seropositive status (75.5% by VNT and 73% by ELISA) on the calving date after vaccination was administered 4–9 months earlier. These results are consistent with previous studies demonstrating that specific antibodies against LSDV in adult cows can persist for a duration of 10–12 months post-vaccination, which is longer than the previously reported seven-month duration (20, 36, 37). Nevertheless, it is recognized that not all animals undergo seroconversion after being vaccinated with live attenuated LSDV vaccines (38). This inconsistency in seroconversion is likely due to the variability of vaccines in inducing antibody immune responses, as demonstrated previously (20, 37, 39). Milovanović et al. (20) indicated that even after booster vaccination, certain cattle remained seronegative. Low responders, characterized by their production of low antibody levels and associated with distinct gene regulation patterns as previously observed in response to the smallpox vaccine (40), may contribute to the explanation of seronegativity being undetectable using currently available tests. Furthermore, the poor immunogenicity of Neethling vaccine strains might lead to low or undetectable antibody levels due to excessive attenuation (41). Consequently, the observation of seronegativity in the present study, as detected by both VNT and ELISA, is not unexpected. Nonetheless, a low antibody response undetected by ELISA or VNT after vaccination might not always indicate a lack of protection in vaccinated cows (42). Furthermore, low vaccine quality due to improper storage or dosage can result in its failure to induce an immune response (38, 43).

Based on the VNT results, it is likely that five VNT-seronegative cows were unable to provide passive immunity to their calves, as indicated by the seronegative VNT results, possibly because seroconversion may not have occurred in these cows. Additionally, they might have developed only low antibody levels or undetectable antibodies, leading to the provision of low-quality colostrum to their calves, resulting in the failure of passive transfer (44). The inability of certain cattle to elicit a humoral response to LSDV can possibly result in the death of young calves due to the absence of colostral antibody production (45). In contrast, the other four VNT-seronegative cows seemed capable of providing passive transfer immunity to their calves through colostrum intake. This circumstance suggests that the absence of neutralizing antibodies in cow serum is linked to the immunoglobulin transfer process for colostrum production in the mammary gland. Consequently, it is probable that neutralizing antibodies are present in colostrum, transferring protective immunity to their calves, as previously reported (46, 47). Recently, Agianniotaki et al. (48) demonstrated that neutralizing antibodies were detected at higher levels in colostrum but found to be present at lower levels in serum. As a result, their calves exhibited LSDV-specific antibodies after consuming colostrum. Regrettably, the presence of neutralizing antibodies in colostrum has not been investigated in this study.

It is well-known that maternally derived antibodies in colostrum are crucial for passive transfer immunity in newborns. High-quality colostrum facilitates the development of innate immunity and antioxidant systems in neonatal calves immediately after birth, ultimately decreasing morbidity and mortality among calves (49). In the present study, VNT-negative calves were detected within the first week after consuming colostrum. As previously discussed, this finding might be due to low-quality colostrum. However, several factors affect successful passive transfer (50, 51), with good colostrum management being a significant contributory factor to this success. Thus, farmers should emphasize intensive colostrum management. In this study, some calves were fed colostrum within 6–12 h. However, it has been suggested that colostrum should be given to calves immediately after birth and within a maximum of 6 h (50). This is because the optimal time for immunoglobulin transfer is 4 h postpartum. If fed after 6 h of birth, the absorption of immunoglobulins will progressively decline (50–52). Importantly, providing adequate colostrum to calves within 6 h after separation from their dams can significantly reduce the risk of the failure of passive transfer (53). However, the majority of calves born to vaccinated cows showed seropositivity to both VNT and ELISA (75.7%) during the first week after birth. Subsequently, antibody levels gradually declined over the course of the study. Detectable maternal antibodies in the calves dropped to 27.03% at 2 months and remained at 13.51% at 3 months of age. It is likely that a number of calves became unprotected after 3 months of age. Accordingly, a previous study has shown that passive transfer antibodies against the homologous Neethling strain of attenuated vaccine in calves can be detected from as early as 3 days after colostrum intake, persisting until they are 3 months old (35.7%) (48). However, these researchers used the Kenyan sheeppox vaccine virus incubated with serum against the homologous Neethling strain virus for VNT. Moreover, similar findings were reported in a study that used a heterologous lumpy skin disease vaccine (Romanian sheeppox vaccine), revealing that passively transferred antibody levels remained protective from 2 to 4 months of age in calves. Interestingly, the duration of the gestation period appears to influence the persistence of passive transfer immunity in newborn calves (54). Nevertheless, it is noteworthy that the maternal immunity derived from ewes vaccinated with the sheeppox vaccine could protect lambs against the virulent sheeppox virus (SPPV) for up to 2 months old (55). Consequently, vaccinating lambs against sheeppox over the age of 2 months seems to be the optimal time frame (55, 56). Although the heterologous virus strain vaccines such as sheeppox or goatpox have been used against LSDV, it is important to emphasize that the homologous Neethling LSDV vaccine has been proven to be more effective and suitable for preventing LSDV in cattle (57, 58).

Maternally, passive immunity can potentially disrupt the development of active immunity in calves up to 6 months old. Therefore, calves born to cows with natural infections or prior vaccinations should be vaccinated at 6 months to ensure sufficient protection (43). According to the manufacturer’s guidelines, vaccination against LSDV should begin at the age of 6 months in calves born to vaccinated cows, followed by an annual booster. In accordance with the European Commission Implementing Decisions, it is recommended that calves born to vaccinated cows be immunized against LSDV at no less than 4 months old (59). However, the results of the present study and a previous study demonstrate a vaccination gap of approximately 3 months in some calves. This suggests that vaccination against LSDV in calves born to vaccinated cows can be initially administered as early as 3 months of age. However, antigenic competition should be investigated if the LSDV vaccine is to be administered at the same time as the first dose of other vaccines, such as the Foot and Mouth Disease (FMD) vaccine (60). Further studies should be conducted to challenge calves with highly virulent LSDV field strains to elucidate the duration of protective passive immunity. Moreover, it would be valuable to assess potential vaccination protocols for calves as young as 2 months old, as suggested by Agianniotaki et al. (48). This would help specify the precise duration of maternal immunity and assess whether low neutralizing antibody levels affect vaccine efficacy.

The maternal passive transfer immunity against the homologous LSDV can persist in some calves born to vaccinated cows at the age of 3 months. This finding provides insight into the immunity status of newborn calves under field conditions on dairy farms in Thailand. This study also reflects the colostrum management on each farm under field conditions. The findings reported here should be useful in the design of the LSDV vaccination program for calves born to vaccinated cows in Thailand and worldwide.

This study evaluated the accuracy of an in-house ELISA test and VNT assay, WOAH-validated assay. A single population model was chosen for Bayesian analysis because the animals were raised in the same region where there was no difference in management practices and the environment. Thus, it was reasonable to expect that all animals were considered as one population, as assumed in previous studies (22, 33, 61). A substantial agreement between the outcomes of both serological tests suggests that their application as serial tests would enhance to increase the overall specificity or performance of LSD testing (22).

The current study reported fairly good Se in the in-house ELISA test; however, it was lower than a previous study, possibly due to the previous study being performed in LSDV-infected adult dairy cattle that may have a better immune response than dairy calves born from the vaccinated dams under study. Furthermore, several studies have reported that adult cattle showed higher seropositivity than young cattle (62, 63). However, the estimated Se for the in-house ELISA test was slightly higher than its prior value. Likewise, posterior estimates for the Se and Sp of the VNT assay were high levels and close to their priors. This finding agrees with previous studies and suggests that the prior selection was appropriate for the observed data (18, 21, 22). In contrast, a study in Ethiopia reported a low Se of VNT assay ranging between 70 and 80% when implemented on a cattle population with unknown disease status (18). However, a study or information on the characteristics of in-house ELISA and VNT tests lacks sufficient information for the prior estimates and narrow priors of both Se and Sp of the tests. This may influence posterior distributions or the convergence of the final model. Furthermore, the low the cut-off of the in-house ELISA, as described previously (22), may impact the true immunity status and potentially lead to more seropositive or false-seropositive results. Consequently, this adjustment may affect the overall performance of the tests. Establishing the appropriate cut-off value may require testing a substantial number of the true uninfected and the true infected animals to ensure accuracy that will stand the test of time (64).

The posterior estimate for true disease prevalence was lower than in a previous study. This finding might be due to a difference in the cattle sample. This study was performed on healthy calves born from vaccinated cows, whereas the previous study was performed on an infected herd, including infected and uninfected animals, during LSD outbreaks (22). Therefore, the true disease prevalence in this study was lower than expected.

## Conclusion

The present study offers information on the persistent duration of maternally passive immunity against LSDV in calves born from vaccinated cows. Neutralizing antibodies were detected in most calves and declined in a few months. This finding suggests the implementation of a suitable vaccination protocol for dairy calves in this area. Additionally, the performance of the virus neutralization test and an in-house ELISA test was estimated, revealing that the Se and Sp of both serological tests were similar. This suggests that the ELISA test can be used as an ancillary test for LSDV immune response detection to improve the efficiency of LSDV-antibody monitoring.

## Data availability statement

The original contributions presented in the study are included in the article/Supplementary material, further inquiries can be directed to the corresponding author.

## Ethics statement

The animal studies were approved by the Animal Care and Use Committee (FVM-ACUC), Faculty of Veterinary Medicine, Chiang Mai University (Ref. No. S8/2565). The studies were conducted in accordance with the local legislation and institutional requirements. Written informed consent was obtained from the owners for the participation of their animals in this study.

## Author contributions

AR: Conceptualization, Data curation, Formal Analysis, Methodology, Project administration, Supervision, Validation, Visualization, Writing – original draft, Writing – review & editing. WM: Data curation, Methodology, Writing – review & editing. KS: Methodology, Validation, Writing – review & editing. AM: Methodology, Validation, Writing – review & editing. PV: Conceptualization, Writing – review & editing. KB: Methodology, Validation, Writing – review & editing. KP: Methodology, Validation, Writing – review & editing. VP: Formal Analysis, Supervision, Visualization, Writing – review & editing. KK: Conceptualization, Formal Analysis, Supervision, Visualization, Writing – review & editing. NS: Conceptualization, Formal Analysis, Funding acquisition, Methodology, Supervision, Validation, Visualization, Writing – review & editing. TS: Conceptualization, Data curation, Formal Analysis, Methodology, Supervision, Validation, Visualization, Writing – original draft, Writing – review & editing.
