# Supplementary material for: Persistence of passive immunity in calves receiving colostrum from cows vaccinated with a live attenuated lumpy skin disease vaccine and the performance of serological tests
Source: Front Vet Sci. 2024 May 21;11:1303424. doi: 10.3389/fvets.2024.1303424 (PMC11148353; doi:10.3389/fvets.2024.1303424)
Supplement: Supplementary file 1 [file Data_Sheet_1.PDF]

Supplementary 1: Two conditionally dependent tests with one population model

```
library(rjags)
library(R2jags)
data <- list( y2 = c(85,8,23,106), n = 222)

Onepop.sen.model <- function(){

y2[1:4] ~ dmulti(q[1:4], n)
q[1] <- pi*(InH_Se*VN_Se+covD1)+(1-pi)*((1-InH_Sp)*(1-VN_Sp)+covD0)
q[2] <- pi*(InH_Se*(1-VN_Se)-covD1)+(1-pi)*((1-InH_Sp)*VN_Sp-covD0)
q[3] <- pi*((1-InH_Se)*VN_Se-covD1)+(1-pi)*(InH_Sp*(1-VN_Sp)-covD0)
q[4] <- pi*((1-InH_Se)*(1-VN_Se)+covD1)+(1-pi)*(InH_Sp*VN_Sp+covD0)

covD1~dunif(a1,b1)
covD0~dunif(a2,b2)
a1<-(InH_Se- 1)*(1-VN_Se)
b1<-min(InH_Se,VN_Se)-(InH_Se*VN_Se)
a2<-(InH_Sp- 1)*(1-VN_Sp)
b2<-min(InH_Sp,VN_Sp)-(InH_Sp*VN_Sp)

InH_Se ~ dbeta(50.14,3.59)
InH_Sp ~ dbeta(25.48,3.72)

VN_Se ~ dbeta(69.42,15.01)
VN_Sp ~ dbeta(100.00,4.06)

pi ~ dbeta(8.90,5.07)}

parameters <- c("InH_Se","VN_Se","InH_Sp","VN_Sp","pi", "covD1","covD0")
```

```
Int1<-list(InH_Se = 0.9, VN_Se = 0.8,InH_Sp = 0.8, VN_Sp = 0.8, pi = 0.4, covD1 =
0.000001,covD0 = 0.0000001)

Int2<-list(InH_Se = 0.8, VN_Se = 0.7,InH_Sp = 0.6, VN_Sp = 0.8, pi = 0.2, covD1 =
0.0000001,covD0 = 0.000001)

Int3<-list(InH_Se = 0.7, VN_Se = 0.9,InH_Sp = 0.7, VN_Sp = 0.8, pi = 0.6, covD1 =
0.000001,covD0 = 0.00000001)

#Int3<-listlist(beta0 = 1, beta1 = 2, sigma = 3)

Ints <-list(Int1,Int2,Int3)

set.seed(1234);date()

jagsfit12 <- jags(data=data ,inits=Ints, n.chains=3,

               parameters.to.save=parameters,

               model.file=Onepop.sen.model,

               n.thin=2,n.burnin = 10000,n.iter=100000);date()

# display the output

print(jagsfit12)
```
