# Supplementary material for: Persistence of passive immunity in calves receiving colostrum from cows vaccinated with a live attenuated lumpy skin disease vaccine and the performance of serological tests
Source: Front Vet Sci. 2024 May 21;11:1303424. doi: 10.3389/fvets.2024.1303424 (PMC11148353; doi:10.3389/fvets.2024.1303424)
Supplement: Supplementary file 2 [file Data_Sheet_2.PDF]

Supplementary 2: Checking of convergence of two conditionally dependent tests with one population model

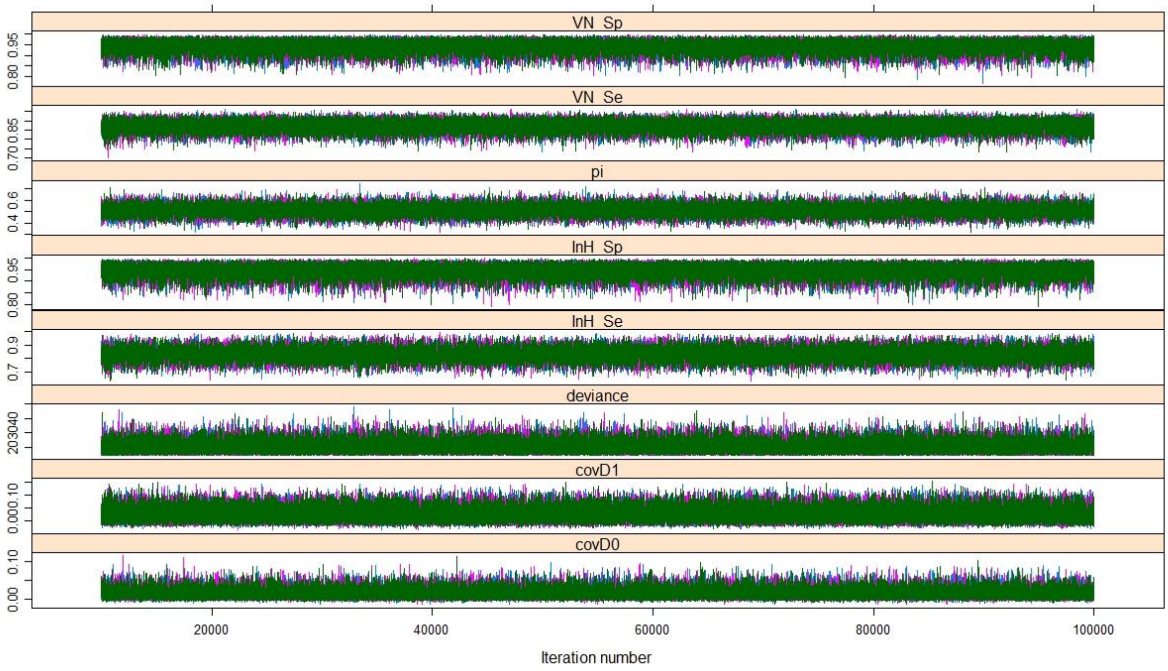

Figure 1 Trace plots of two conditionally dependent tests with one population model

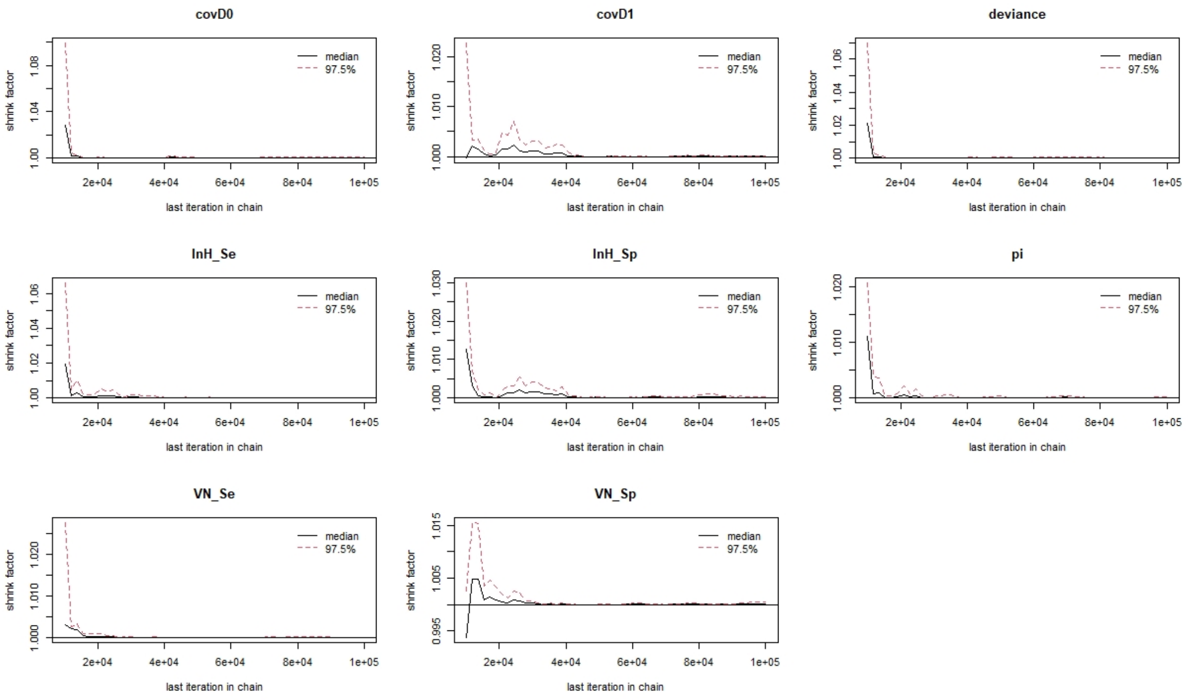

Figure 2 Gelman-Rubin diagnostic plots of two conditionally dependent tests with one population model
